# Comprehensive Bioinformatics Analysis of Glycosylation-Related Genes and Potential Therapeutic Targets in Colorectal Cancer

**DOI:** 10.3390/ijms26041648

**Published:** 2025-02-14

**Authors:** Po-Kai Chuang, Kai-Fu Chang, Chih-Hsuan Chang, Ting-Yu Chen, Yueh-Jung Wu, Hui-Ru Lin, Chi-Jen Wu, Cheng-Chun Wu, Yu-Cheng Ho, Chih-Chun Lin, Chien-Han Yuan, Chih-Yang Wang, Yung-Kuo Lee, Tung-Yuan Chen

**Affiliations:** 1 Institute of Biomedical Sciences, National Sun Yat-sen University, Kaohsiung 80424, Taiwan; pkchuang@mail.nsysu.edu.tw (P.-K.C.); abstyle0204@gmail.com (C.-H.C.); aqw373885@gmail.com (T.-Y.C.); 2 Medical Laboratory, Medical Education and Research Center, Kaohsiung Armed Forces General Hospital, Kaohsiung 80284, Taiwan; zjack5109@gmail.com (K.-F.C.); han86449@gmail.com (C.-H.Y.); 3 Division of Colorectal Surgery, Department of Surgery, Kaohsiung Armed Forces General Hospital, Kaohsiung 80284, Taiwan; allenyjw28@gmail.com; 4 Institute of Medical Science and Technology, National Sun Yat-sen University, Kaohsiung 80424, Taiwan; linlulu0805@gmail.com; 5 Nursing Department, Kaohsiung Armed Forces General Hospital, Kaohsiung 80284, Taiwan; iris189212@gmail.com; 6 College of Nursing, Kaohsiung Medical University, Kaohsiung 80708, Taiwan; 7 School of Medicine, College of Medicine, I-Shou University, Kaohsiung 82445, Taiwan; chengchunwu@isu.edu.tw (C.-C.W.); ycho@isu.edu.tw (Y.-C.H.); 8 Department of Physical Therapy, I-Shou University, Kaohsiung 824005, Taiwan; chihchunlin@isu.edu.tw; 9 Department of Otolaryngology, Kaohsiung Armed Forces General Hospital, Kaohsiung 80284, Taiwan; 10 Department of Otolaryngology, National Defense Medical Center, Taipei 11490, Taiwan; 11 Ph.D. Program for Cancer Molecular Biology and Drug Discovery, College of Medical Science and Technology, Taipei Medical University, Taipei 11031, Taiwan; chihyang@tmu.edu.tw; 12 Graduate Institute of Cancer Biology and Drug Discovery, College of Medical Science and Technology, Taipei Medical University, Taipei 11031, Taiwan; 13 Division of Experimental Surgery Center, Department of Surgery, Tri-Service General Hospital, National Defense Medical Center, Taipei 11490, Taiwan

**Keywords:** colorectal cancer, glycosylation, prognostic model, machine learning, immune microenvironment, bioinformatics, drug sensitivity analysis

## Abstract

Colorectal cancer (CRC) is a leading cause of cancer-related deaths worldwide, characterized by high incidence and poor survival rates. Glycosylation, a fundamental post-translational modification, influences protein stability, signaling, and tumor progression, with aberrations implicated in immune evasion and metastasis. This study investigates the role of glycosylation-related genes (Glycosylation-RGs) in CRC using machine learning and bioinformatics. Data from The Cancer Genome Atlas (TCGA) and the Molecular Signatures Database (MSigDB) were analyzed to identify 67 differentially expressed Glycosylation-RGs. These genes were used to classify CRC patients into two subgroups with distinct survival outcomes, highlighting their prognostic value. Weighted gene coexpression network analysis (WGCNA) revealed key modules associated with CRC traits, including pathways like glycan biosynthesis and PI3K–Akt signaling. A machine-learning-based prognostic model demonstrated strong predictive performance, stratifying patients into high- and low-risk groups with significant survival differences. Additionally, the model revealed correlations between risk scores and immune cell infiltration, providing insights into the tumor immune microenvironment. Drug sensitivity analysis identified potential therapeutic agents, including Trametinib, SCH772984, and Oxaliplatin, showing differential efficacy between risk groups. These findings enhance our understanding of glycosylation in CRC, identifying it as a critical factor in disease progression and a promising target for future therapeutic strategies.

## 1. Introduction

Colorectal cancer (CRC) is one of the leading causes of cancer-related mortality worldwide, characterized by high incidence and poor survival in advanced stages [1,2,3]. Despite significant advances in therapeutic strategies, the prognosis for CRC patients remains suboptimal, largely due to tumor heterogeneity, delayed detection, and the limited sensitivity of current diagnostic tools [4,5]. These challenges complicate early intervention and necessitate the development of more precise diagnostic approaches. Understanding these mechanisms is essential for improving early detection and developing personalized therapeutic approaches [5,6,7].

Glycosylation is a fundamental post-translational modification that plays a critical role in protein folding, stability, and signaling [8,9]. Aberrant glycosylation has been implicated in various biological processes, including tumor progression, immune evasion, and metastasis [10,11]. It affects cell–cell communication, alters the tumor microenvironment, and enables cancer cells to evade immune surveillance [10,12,13]. Recent studies have highlighted its potential as a biomarker for cancer prognosis and a target for therapeutic interventions [14,15]. However, the specific contributions of glycosylation-related genes (Glycosylation-RGs) to CRC remain inadequately explored.

The dynamic interplay between glycosylation and oncogenic pathways has garnered attention, particularly in its role in modulating epithelial–mesenchymal transition (EMT), angiogenesis, and drug resistance [16,17]. Furthermore, glycosylation patterns are known to vary across different stages of cancer progression, presenting an opportunity to refine staging and risk stratification for CRC patients [18,19].

Advancements in high-throughput sequencing technologies and bioinformatics have provided an unprecedented opportunity to investigate the molecular landscape of CRC [20,21,22]. Computational methods, including machine learning algorithms, enable the integration of multi-omics data to uncover novel biomarkers and predict patient outcomes with high accuracy [23,24]. Specifically, machine learning models can analyze complex datasets to predict patient-specific responses to therapies, identify early-stage biomarkers with improved sensitivity, and stratify patients into risk groups to guide personalized treatment strategies [21,25,26]. In recent years, machine learning has emerged as a powerful tool for predicting cancer prognosis, particularly in the context of biochemical recurrence. Some studies have employed the weighted gene coexpression network analysis (WGCNA) method [27,28] with the Gene Expression Omnibus (GEO) and The Cancer Genome Atlas (TCGA) to identify and filter differentially expressed genes that are significantly associated with development of cancer progression and prognosis [29]. Integrating gene expression data with clinical outcomes can reveal critical pathways and identify prognostic biomarkers [30,31]. This study focuses on the systematic analysis of glycosylation-related genes in CRC, aiming to elucidate their role in tumor progression and the tumor immune microenvironment [19,32,33,34].

Here, we conducted a comprehensive analysis using publicly available datasets to identify glycosylation-related genes associated with CRC. We explored their differential expression, constructed a prognostic risk model, and examined their correlation with immune cell infiltration. Our findings provide valuable insights into the functional and clinical significance of glycosylation-related genes in CRC, paving the way for potential therapeutic strategies.

## 2. Results

### 2.1. Identification of Differentially Expressed Overlapping Glycosylation-Related Genes

**Figure 1:**
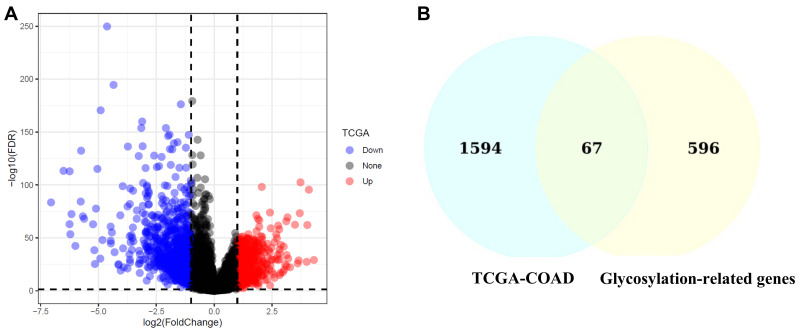
Identification of differentially expressed glycosylation-related genes in CRC. (**A**) Volcano plot displaying the distribution of differentially expressed genes in the TCGA-COAD dataset. Blue dots indicate downregulated genes, red dots indicate upregulated genes, and black dots represent non-significant genes. The *x*-axis shows log2 (fold change), while the *y*-axis shows −log10 (FDR). (**B**) Venn diagram illustrating the overlap between glycosylation-related genes and differentially expressed genes in TCGA-COAD. The intersection identifies 67 overlapping genes critical for further analysis.

To identify glycosylation-related genes involved in CRC, we analyzed TCGA datasets and identified 1661 differentially expressed genes (DEGs). Using the MSigDB database, 663 glycosylation-related genes were retrieved. Among these, 67 genes were identified as differentially expressed (Supplementary File S1) and overlapping with glycosylation-related genes. The volcano plot shows the distribution of DEGs, with significant upregulation and downregulation patterns (Figure 1A). The Venn diagram highlights the intersection between glycosylation-related genes and DEGs, underscoring the relevance of these genes in CRC (Figure 1B).

Functional enrichment analysis of the 67 overlapping genes revealed significant involvement in biological pathways, such as glycan biosynthesis, N-glycan modification, and cell adhesion processes. These pathways are known to contribute to tumor progression and metastasis, suggesting their critical role in CRC.

### 2.2. Identification of Glycosylation-Related Subgroups

**Figure 2:**
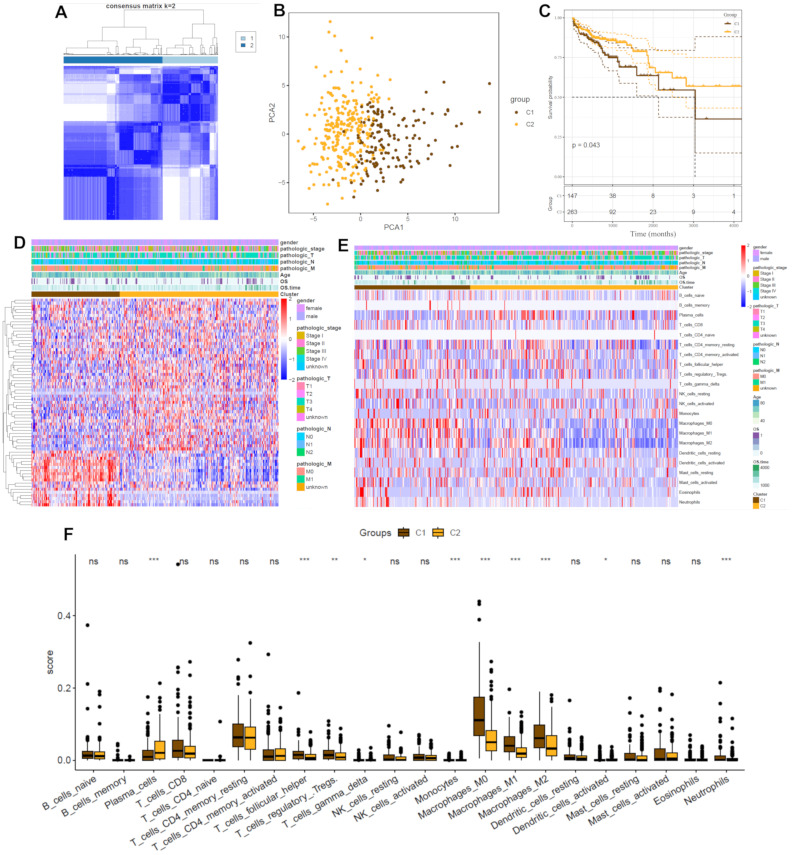
Identification of glycosylation-related subgroups in CRC. (**A**) Consensus matrix heatmap demonstrating optimal clustering into two subgroups (C1 and C2) using glycosylation-related genes. (**B**) Principal component analysis (PCA) showing the separation between subgroups C1 and C2. (**C**) Kaplan–Meier survival curves illustrating overall survival differences between the subgroups, with subgroup C1 showing poorer survival outcomes (*p* = 0.043). The *x*-axis represents time in months. (**D**) Heatmap displaying glycosylation-related gene expression in subgroups C1 and C2, alongside clinicopathological characteristics. (**E**) Heatmap depicting immune cell infiltration patterns in the two subgroups. (**F**) Boxplots showing significant differences in the abundance of specific immune cell types between subgroups C1 and C2 (e.g., macrophages M0, macrophages M1, and macrophages M2, ns: not significant, * *p* < 0.05, ** *p* < 0.01, and *** *p* < 0.001).

Unsupervised consensus clustering was performed using the expression profiles of the 67 glycosylation-related genes, classifying CRC patients into two distinct subgroups, C1 and C2. The optimal number of clusters (k = 2) was determined using the consensus cumulative distribution function (CDF) (Figure 2A). Principal component analysis (PCA) confirmed the clear separation between the two subgroups, indicating distinct molecular characteristics (Figure 2B). Kaplan–Meier survival analysis revealed that patients in subgroup C1 exhibited significantly poorer survival outcomes compared to those in subgroup C2 (*p* < 0.001, Figure 2C). The heatmap of gene expression further illustrated distinct glycosylation-related gene expression patterns between the two subgroups, correlating with clinical traits such as tumor stage and metastasis (Figure 2D). A heatmap illustrating the differences in immune cell composition between the subgroups highlights variations in the infiltration of key immune cell types (Figure 2E). Figure 2F further quantifies these differences through boxplots, demonstrating statistically significant variations in specific immune cell populations, such as macrophages (M0, M1, and M2), dendritic cells, and Tregs between the subgroups. These findings provide insights into the tumor immune microenvironment and suggest that glycosylation-related molecular subtypes may be associated with distinct immune responses, which could have implications for immunotherapy strategies in CRC.

### 2.3. Identification of Highly Correlated Gene Modules in CRC

**Figure 3:**
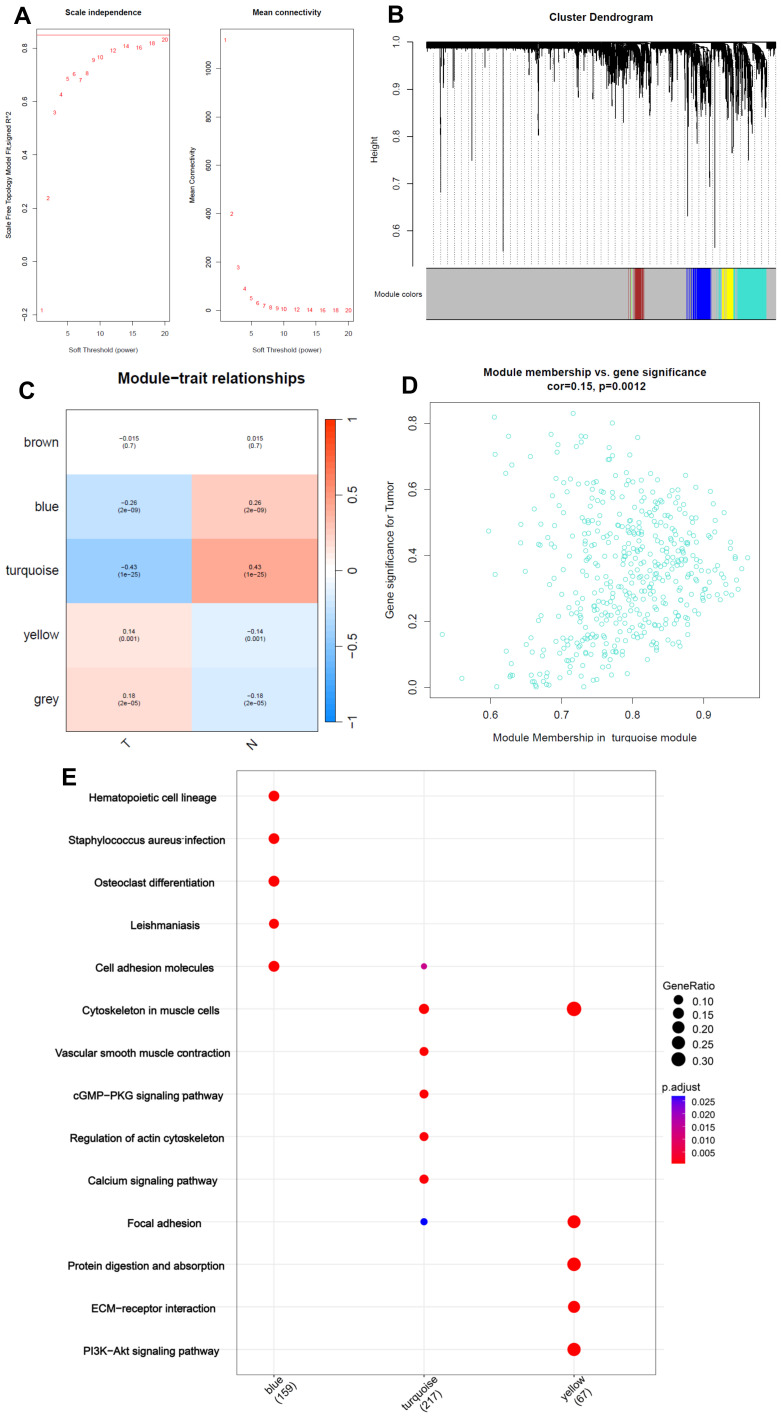
Identification of highly correlated gene modules using weighted gene coexpression network analysis (WGCNA). (**A**) Soft thresholding power determination for constructing a scale-free network, showing the scale-free topology fit index and mean connectivity for different power values. (**B**) Cluster dendrogram of glycosylation-related genes grouped into distinct modules, with colors representing module membership. (**C**) Module–trait relationships illustrating correlations between modules (e.g., turquoise, blue, brown) and clinical traits, such as tumor presence. (**D**) Scatter plot showing the correlation between module membership and gene significance in the turquoise module, which is highly associated with CRC. (**E**) KEGG pathway enrichment analysis for genes in the turquoise module, highlighting pathways such as “focal adhesion,” “PI3K–Akt signaling,” and “ECM–receptor interaction.

Using WGCNA, we constructed a gene coexpression network to identify modules associated with CRC traits. The turquoise module exhibited the strongest correlation with tumor traits and included several glycosylation-related genes (Figure 3A–D). Functional enrichment analysis of the turquoise module genes highlighted pathways such as cGMP–PKG signaling, cytoskeleton in muscle cells, extracellular matrix organization, and PI3K–Akt signaling (Figure 3E). Gene significance and module membership analysis revealed key glycosylation-related genes. These genes are known to influence tumor invasiveness and immune suppression [35].

### 2.4. Identification of Glycosylation-Related Gene Clusters

**Figure 4:**
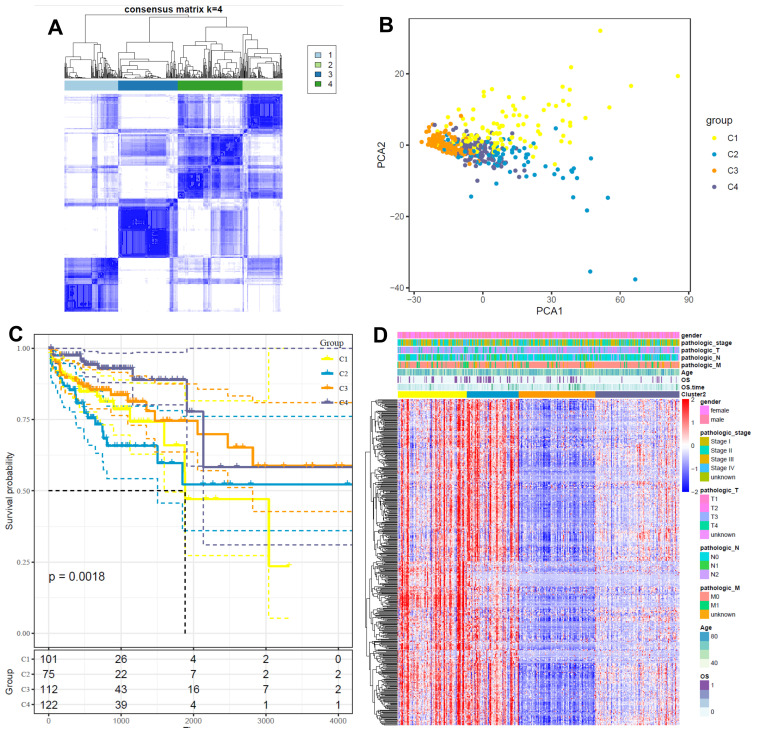
Identification of glycosylation-related gene clusters in CRC. (**A**) Consensus matrix heatmap defining four gene clusters (C1, C2, C3, and C4) based on glycosylation-related gene expression. (**B**) Principal component analysis (PCA) visualizing the separation of samples into four clusters. (**C**) Kaplan–Meier survival curves showing significant differences in overall survival among the four clusters (*p* = 0.0018), with cluster C1 demonstrating the poorest survival. (**D**) Heatmap depicting the expression patterns of glycosylation-related genes across the four clusters, alongside associated clinicopathological characteristics.

Consensus clustering was extended to identify four distinct gene clusters (C1–C4) using glycosylation-related genes (Figure 4A). PCA showed clear separation of these clusters (Figure 4B), while Kaplan–Meier analysis revealed significant survival differences, with cluster C1 associated with the poorest prognosis (Figure 4C). A heatmap illustrates the differential expression of glycosylation-related genes across clusters, along with clinicopathological annotations (Figure 4D).

### 2.5. Construction of a Glycosylation-Related Prognostic Risk Model

**Figure 5:**
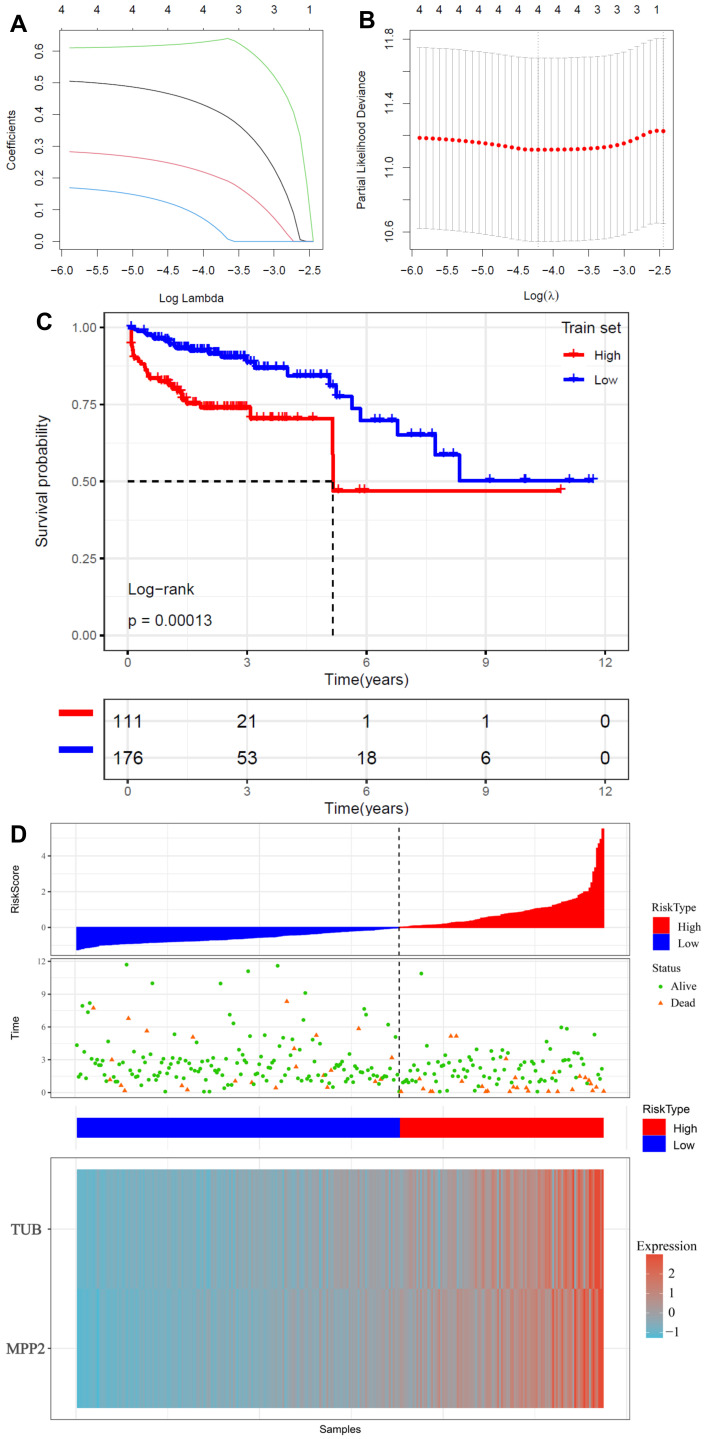
Construction of a glycosylation-related prognostic risk model for CRC. (**A**) Coefficient profiles of selected genes during LASSO regression. Different-colored lines represent individual genes. (**B**) Partial likelihood deviance plot to determine the optimal lambda value for the model, with the minimum value marked by a dotted line. (**C**) Kaplan–Meier survival curves showing a significant difference in overall survival between high-risk (red) and low-risk (blue) groups in the training dataset (*p* = 0.00013). (**D**) Distribution of risk scores, survival status, and gene expression profiles in the high- and low-risk groups. The top panel shows risk scores, the middle panel displays survival outcomes (green dots: alive; orange triangles: deceased), and the bottom heatmap illustrates the expression patterns of selected prognostic genes.

LASSO regression and Cox proportional hazards analysis were employed to construct a prognostic risk model based on glycosylation-related genes. Four genes (TUB, TCF7L1, MPP2, and TMEM59L) were selected for the final model, which stratified patients into high- and low-risk groups (Figure 5A,B). Kaplan–Meier survival analysis demonstrated that high-risk patients had significantly worse survival outcomes (*p* < 0.00013, Figure 5C) compared to those in the low-risk group. Furthermore, the distribution of risk scores and survival outcomes is shown in Figure 5D. The top panel displays the stratification of patients by risk score, while the middle panel illustrates survival status, where green dots denote living patients and orange triangles represent deceased individuals. The heatmap in the bottom panel highlights the expression profiles of the selected prognostic genes across the high- and low-risk groups, demonstrating distinct expression patterns correlating with risk stratification.

### 2.6. Validation of the Glycosylation-Related Prognostic Risk Model

**Figure 6:**
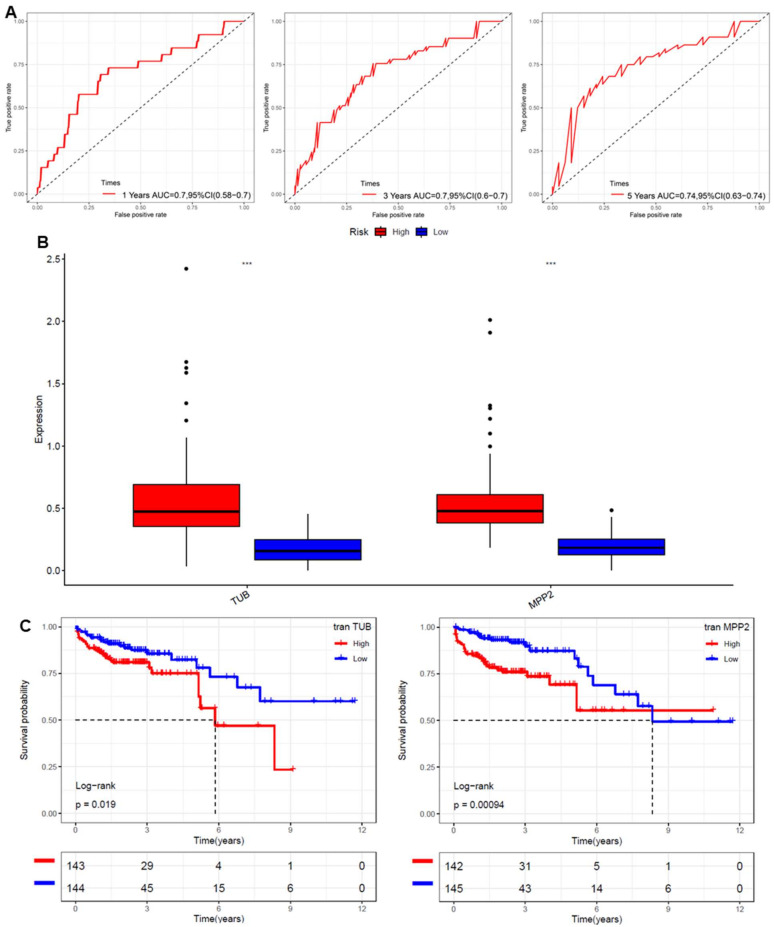
Validation of the glycosylation-related prognostic risk model for CRC. (**A**) Time-dependent ROC curves for predicting overall survival at 1, 3, and 5 years, showing AUC values of 0.7, 0.7, and 0.74, respectively. (**B**) Boxplots comparing the expression levels of the key prognostic genes (TUB and MPP2) between high- and low-risk groups, indicating significant differences (*** *p* < 0.001). (**C**) Kaplan–Meier survival curves for individual genes TUB (left) and MPP2 (right), with high expression associated with poorer overall survival (*p* = 0.019 and *p* = 0.00094, respectively).

The glycosylation-related prognostic risk model for CRC was validated using time-dependent ROC curves, as shown in Figure 6A. The model demonstrated good predictive accuracy for overall survival, with AUC values of 0.7, 0.7, and 0.74 for 1-year, 3-year, and 5-year survival, respectively. Figure 6B presents boxplots comparing the expression levels of the key prognostic genes, TUB and MPP2, between high- and low-risk groups. Both genes were significantly overexpressed in the high-risk group (*** *p* < 0.001), supporting their relevance to the risk stratification. Kaplan–Meier survival analysis for individual genes (Figure 6C) revealed that high expression levels of TUB and MPP2 were significantly associated with poorer overall survival (log-rank *p* = 0.019 and *p* = 0.00094, respectively). These findings further confirm the robustness and prognostic value of the model in predicting CRC outcomes.

### 2.7. Correlation Between Risk Scores and Immune Cell Infiltration

**Figure 7:**
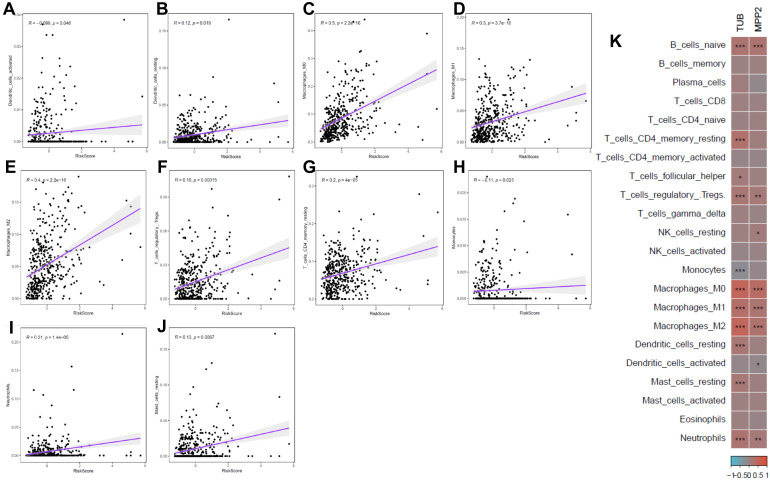
Correlation between immune cell infiltration and glycosylation-related risk scores in CRC. (**A**–**J**) Scatter plots showing significant correlations between the glycosylation-related risk score and the infiltration levels of various immune cell types, including activated (**A**) dendritic cells, (**B**) resting dendritic cells, (**C**) macrophages M0, (**D**) macrophages M1, (**E**) macrophages M2, (**F**) regulatory T cells, (**G**) resting memory CD4+ T cells, (**H**) monocytes, (**I**) neutrophils, and (**J**) resting mast cells. Each purple line indicates the linear regression trend, with shaded areas representing the 95% confidence interval. (**K**) Heatmap illustrating the relationship between the expression of key prognostic genes (TUB and MPP2) and immune cell infiltration. Red indicates positive correlation, and blue indicates negative correlation (* *p* < 0.05, ** *p* < 0.01, and *** *p* < 0.001).

CIBERSORT analysis revealed significant correlations between glycosylation-related risk scores and immune cell infiltration. High-risk patients exhibited increased infiltration of suppressive immune cells, such as regulatory T cells and M2 macrophages, alongside reduced activation of cytotoxic T cells and NK cells. These findings suggest that aberrant glycosylation may contribute to an immunosuppressive tumor microenvironment (Figure 7A–K).

Heatmap analysis further illustrated the relationship between key glycosylation-related genes and immune cell infiltration, highlighting their potential role in modulating immune escape mechanisms in CRC. These findings provide a comprehensive view of the molecular and immunological landscape shaped by glycosylation in CRC progression.

### 2.8. Drug Sensitivity Prediction in High- and Low-Risk Groups

To investigate potential therapeutic strategies, drug sensitivity analysis was conducted using the oncoPredict algorithm. The analysis identified the top five drugs with the largest and smallest differences in predicted sensitivity between high-risk and low-risk groups.

**Figure 8:**
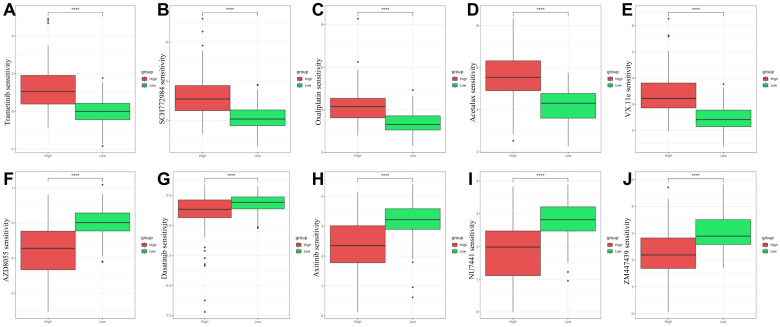
Drug sensitivity analysis (IC50) of high- and low-risk groups predicted by oncoPredict. Boxplots display the top five drugs with the largest differences in predicted sensitivity between high-risk (red) and low-risk (green) groups and the top five drugs with the smallest differences. (**A**) Trametinib, (**B**) SCH772984, (**C**) oxaliplatin, (**D**) Acetalax, and (**E**) VX-11e are the top five drugs with significantly higher sensitivity in the low-risk group. Conversely, (**F**) AZD8055, (**G**) doxorubicin, (**H**) axitinib, (**I**) NU7441, and (**J**) ZM447439 showed lower differences in sensitivity between the groups.

The low-risk group exhibited significantly higher sensitivity to drugs targeting the MAPK pathway, including trametinib (Figure 8A) and SCH772984 (Figure 8B), as well as the chemotherapy agent oxaliplatin (Figure 8C). Acetalax, a BCL-2 inhibitor, and VX-11e, an ERK inhibitor, also showed significantly enhanced efficacy in the low-risk group (Figure 8D,E). These findings highlight the potential for these agents to provide improved therapeutic outcomes in patients with favorable risk profiles.

In contrast, the drugs AZD8055, doxorubicin, axitinib, NU7441, and ZM447439 demonstrated higher predicted sensitivity between high-risk groups (Figure 8F–J). These results suggest that these agents may exhibit significant stratified efficacy in CRC treatment based on the identified glycosylation-related risk model. These findings provide a basis for further exploring the translational application of these drugs in stratified CRC treatment, enabling personalized therapeutic strategies to improve patient outcomes.

## 3. Discussion

This study systematically analyzed glycosylation-related genes in colorectal cancer (CRC) and established their potential role in tumor progression, prognosis, and the tumor immune microenvironment. Differential expression analysis revealed that 67 glycosylation-related genes were significantly dysregulated in CRC, suggesting their functional involvement in oncogenic pathways (Figure 1A,B) [36,37].

The identification of CRC subgroups based on glycosylation-related gene expression profiles highlighted distinct clinical and molecular characteristics. Subgroup C2 demonstrated significantly worse survival outcomes compared to C1, emphasizing the prognostic value of glycosylation-related genes in CRC (Figure 2A–D). These findings align with previous reports implicating glycosylation in tumor heterogeneity and aggressiveness [11,38]. Notably, these subgroups provide a framework for personalized treatment strategies, underscoring the potential of molecular classification in optimizing patient management [39].

Using WGCNA, the turquoise module was identified as a key coexpression network associated with CRC traits. Functional enrichment analysis highlighted the role of this module in critical pathways such as glycan biosynthesis, focal adhesion, and PI3K–Akt signaling, supporting its biological relevance in CRC progression (Figure 3A–E). The strong correlation between these pathways and tumor invasiveness, metastasis, and immune evasion further validates the significance of glycosylation in the broader context of cancer biology [40].

The construction of a prognostic risk model based on glycosylation-related genes represents a significant advance in CRC management. High-risk patients identified by the model exhibited markedly poorer survival, and the model demonstrated robust predictive performance across multiple datasets (Figure 5A–D, Figure 6A–C). These results underscore the clinical utility of glycosylation-related genes as prognostic biomarkers. Additionally, the machine learning approach used to build this model exemplifies how computational tools can optimize biomarker selection and stratification processes, ensuring higher accuracy and reproducibility [41,42].

Furthermore, this study revealed significant associations between glycosylation-related risk scores and immune cell infiltration, including macrophages, regulatory T cells, and neutrophils. These findings suggest that glycosylation may influence the tumor immune microenvironment, offering new insights into potential immunotherapeutic strategies (Figure 7A–K). Specifically, glycosylation patterns could affect the expression of immune checkpoints or the recruitment of suppressive immune cells, which are critical in shaping the tumor microenvironment [43].

The translational potential of targeting glycosylation pathways in CRC treatment is immense. Aberrant glycosylation could serve as both a diagnostic marker and a therapeutic target. For instance, glycosylation inhibitors or monoclonal antibodies targeting glycan-modified proteins might enhance the efficacy of existing therapies, including immune checkpoint inhibitors and chemotherapy [44]. In addition, the interplay between glycosylation and immune cell recruitment presents opportunities to modulate the tumor immune microenvironment, potentially enhancing anti-tumor immunity. The integration of glycosylation-targeted therapies with existing treatment regimens could lead to synergistic effects, improving patient outcomes [45].

Machine learning played a pivotal role in this study, enabling the integration of high-dimensional data and the identification of robust biomarkers. Its application to risk stratification and survival prediction highlights its value in precision oncology. Future research should incorporate additional omics layers, such as metabolomics and proteomics, to develop more comprehensive models. Integrating these models with clinical data could further refine prognostic predictions and guide individualized therapeutic strategies. Moreover, exploring deep learning techniques could further enhance the predictive capabilities of models used for biomarker discovery [46,47].

Despite its contributions, this study has limitations including overfitting due to small sample sizes, lack of specific gene markers for CRC prognosis, and insufficient external validation. Validating the glycosylation-related prognostic risk model’s predictive power using in vitro and in vivo validation would indeed provide direct experimental confirmation. The reliance on publicly available datasets may introduce biases related to cohort selection and data quality. Experimental validation of identified biomarkers in larger, more diverse cohorts is necessary to confirm their clinical utility [48]. Additionally, while the prognostic model showed high predictive accuracy, external validation in prospective studies is required to establish its generalizability. A further limitation is the lack of mechanistic studies to directly validate the functional role of glycosylation in CRC progression and immune modulation, which should be a focus for future research [49].

The integration of drug sensitivity analysis represents a significant expansion by this study, providing novel insights into potential therapeutic strategies for CRC patients stratified by glycosylation-related risk groups. Using oncoPredict, we identified five drugs (trametinib, SCH772984, oxaliplatin, Acetalax, and VX-11e) with significant differential sensitivity between high- and low-risk groups (Figure 8A–E). These findings underscore the translational potential of tailoring therapeutic approaches to molecularly defined subgroups. The higher sensitivity of the low-risk group to trametinib and SCH772984 highlights the relevance of targeting the MAPK signaling pathway, which is frequently dysregulated in CRC. This pathway, activated downstream of cGMP-dependent protein kinases (PKG) in response to cGMP signaling (Figure 3E), has been implicated in increasing tumor cell stemness and metastasis [50]. The cGMP–PKG–MAPK cascade underscores a critical molecular mechanism by which tumor progression and therapy resistance may be mediated, providing a further rationale for therapeutic intervention in this pathway.

Additionally, the increased efficacy of oxaliplatin in the low-risk group suggests that stratification based on glycosylation-related risk scores may refine patient selection for standard chemotherapy regimens. Acetalax, a BCL-2 inhibitor, demonstrated enhanced sensitivity in the low-risk group, pointing to the potential of apoptosis-targeting therapies in this cohort. Similarly, VX-11e further supports the utility of targeting the ERK pathway. In contrast, drugs such as AZD8055, doxorubicin, axitinib, NU7441, and ZM447439 showed lower differences in predicted sensitivity between high- and low-risk groups (Figure 8F–J). This suggests that these agents may not exhibit substantial stratified efficacy in CRC treatment based on glycosylation-related risk models. The identification of these drugs also underscores the specificity of certain therapeutic agents to molecularly distinct subgroups.

These drug sensitivity predictions extend the utility of the glycosylation-related prognostic model beyond survival forecasting to therapeutic guidance. By integrating molecular stratification with drug efficacy predictions, this study provides a framework for precision oncology. Future experimental validation of these predictions and exploration of combination therapies targeting glycosylation-related pathways and tumor-specific vulnerabilities are warranted.

In conclusion, our findings highlight the critical role of glycosylation in CRC progression and its potential as a biomarker and therapeutic target. By leveraging machine learning and advanced bioinformatics, this study provides a robust framework for understanding glycosylation’s contributions to CRC and opens avenues for innovative therapeutic interventions. Further experimental and clinical investigations are warranted to fully realize the potential of glycosylation-related therapies in CRC management.

## 4. Materials and Methods

### 4.1. Data Collection and Preprocessing

Gene expression profiles and corresponding clinical data for colorectal cancer (CRC) were downloaded from The Cancer Genome Atlas (TCGA) database. The dataset included RNA-seq data normalized as fragments per kilobase of transcript per million mapped reads (FPKM) values. Clinical information included patient demographics, tumor stage, and survival outcomes.

### 4.2. Identification of Glycosylation-Related Genes (Glycosylation-RGs)

To investigate the role of glycosylation-related genes in CRC, a comprehensive list of glycosylation-related genes was obtained by searching the Molecular Signatures Database (MSigDB; URL: https://www.gsea-msigdb.org/gsea/msigdb/human/collections.jsp) (accessed on 19 August 2024) using the keyword “Glycosylation”. A total of 663 glycosylation-related genes were retrieved for further analysis.

### 4.3. Differential Gene Expression Analysis

Differentially expressed genes (DEGs) between CRC and adjacent normal tissue samples were identified using the “limma” R package. Genes with |log2 fold change| > 1 and adjusted *p*-value < 0.05 were considered significantly differentially expressed. Overlapping genes between DEGs and the 663 glycosylation-related genes were identified using Venn diagram analysis.

### 4.4. Consensus Clustering

Unsupervised consensus clustering was performed using the “ConsensusClusterPlus” R package to classify CRC samples based on the expression profiles of overlapping glycosylation-related genes. The optimal number of clusters was determined using the consensus cumulative distribution function (CDF), which assesses the stability of clusters by evaluating consensus values over repeated iterations. We selected k = 2 as the optimal number of clusters.

### 4.5. Weighted Gene Coexpression Network Analysis (WGCNA)

WGCNA was conducted to construct coexpression networks and identify gene modules associated with CRC traits. A soft thresholding power was selected to achieve scale-free topology, and modules were correlated with clinical traits to identify the most relevant module for downstream analysis.

### 4.6. Prognostic Model Construction and Validation

LASSO regression and multivariate Cox proportional hazards analysis were used to develop a glycosylation-related prognostic risk model. Gene expression profiles and clinical survival data were obtained from The Cancer Genome Atlas (TCGA) dataset, where preprocessing steps included normalization and log2 transformation to reduce batch effects and enhance comparability. In LASSO regression, the optimal λ (lambda) value was selected using 10-fold cross-validation, minimizing the mean squared error to balance model complexity and predictive accuracy. The Cox proportional hazards model was then applied to refine the selection of significant prognostic genes. A risk score formula was then generated based on the expression levels of the four selected genes, weighted by their LASSO-derived coefficients, and patients were stratified into high-risk and low-risk groups using the median risk score as the cutoff. We used the TCGA-CRC dataset for model development and validation. The dataset was randomly split into a training set (70%) and a testing set (30%) to evaluate the generalizability of our model. Model performance was assessed through Kaplan–Meier survival analysis, which demonstrated a significant survival difference between the two groups (*p* < 0.001), and time-dependent receiver operating characteristic (ROC) curves, achieving AUC values of 0.7, 0.7, and 0.74 for 1-year, 3-year, and 5-year survival predictions, respectively.

### 4.7. Immune Infiltration Analysis

The relative abundance of immune cell types in CRC samples was estimated using the CIBERSORT algorithm. Correlation analysis was performed to evaluate the relationship between glycosylation-related risk scores and immune cell infiltration levels. A heatmap was generated to visualize the association between key prognostic genes and immune cell infiltration.

### 4.8. Functional Enrichment Analysis

Gene Ontology (GO) and Kyoto Encyclopedia of Genes and Genomes (KEGG) pathway enrichment analyses were performed for genes in the turquoise module identified by WGCNA. Enrichment analyses were conducted using the “clusterProfiler” R package 4.0, and pathways with adjusted *p*-values < 0.05 were considered significantly enriched.

### 4.9. Drug Sensitivity Analysis

Drug sensitivity predictions were performed using the oncoPredict R package, an extension of the earlier pRRophetic tool developed by Paul Geeleher and colleagues at the University of Minnesota. OncoPredict predicts in vivo or cancer patient drug response based on gene expression data and IC50 values derived from cell line screening data. This package builds on the oncoPredict algorithm [51], which was introduced in 2014 and employs ridge regression to model relationships between baseline gene expression and in vitro drug sensitivity in cell lines [52].

### 4.10. Statistical Analysis

All statistical analyses were performed in R software (version 4.4.1), and a *p*-value < 0.05 was considered statistically significant.

## 5. Conclusions

This study systematically highlights the significance of glycosylation-related genes in colorectal cancer (CRC) progression and prognosis. By integrating bioinformatics and machine learning approaches, we identified key glycosylation-related genes and constructed a robust prognostic risk model capable of stratifying patients into high- and low-risk groups. These findings not only deepen our understanding of the molecular mechanisms underlying CRC but also underscore the pivotal role of glycosylation in modulating the tumor immune microenvironment.

The addition of drug sensitivity analysis further expands the translational potential of this work. By leveraging oncoPredict, we identified five key drugs (trametinib, SCH772984, oxaliplatin, Acetalax, and VX-11e) with significant differences in predicted efficacy between high- and low-risk groups. These findings suggest that the identified risk model can serve not only as a prognostic tool but also as a guide for personalized therapeutic strategies. The low-risk group demonstrated higher sensitivity to targeted agents such as trametinib and SCH772984, emphasizing the relevance of MAPK pathway inhibitors, while also exhibiting enhanced efficacy with standard chemotherapy agents like oxaliplatin. These insights pave the way for integrating molecular stratification with personalized drug selection to optimize CRC treatment outcomes.

Furthermore, the study identified five additional drugs (AZD8055, doxorubicin, axitinib, NU-7441, and ZM447439) that showed lower sensitivity differences between risk groups. While these agents may not have stratified efficacy based on the current risk model, their identification provides additional context for exploring broader therapeutic applications in CRC treatment. The combined insights from prognostic modeling and drug sensitivity analysis highlight the value of integrating molecular biomarkers with therapeutic strategies to refine treatment paradigms.

The study’s results suggest that targeting aberrant glycosylation could hold promise for improving therapeutic strategies, including enhancing the efficacy of immune checkpoint inhibitors and chemotherapy. Furthermore, the integration of glycosylation-related biomarkers into clinical workflows may refine CRC staging, improve early detection, and inform tailored treatment regimens. Future studies should focus on validating these findings in diverse cohorts and experimental settings to fully realize the clinical potential of glycosylation-related therapies in CRC.

## Data Availability

All data involved in this study are available from the corresponding author on request. We employed the Molecular Signatures Database (MSigDB; URL: https://www.gsea-msigdb.org/gsea/msigdb/human/collections.jsp) (accessed on 19 August 2024), using the keyword “Glycosylation” for analysis in this study.

## Supplementary Materials

The supporting information can be downloaded at: https://www.mdpi.com/article/10.3390/ijms26041648/s1.

## Abbreviations

The following abbreviations are used in this manuscript:

CRC	Colorectal cancer
TCGA	The Cancer Genome Atlas
FPKM	Fragments per kilobase of transcript per million mapped
MSigDB	Molecular Signatures Database
WGCNA	Weighted gene coexpression network analysis
CDF	Consensus cumulative distribution function
ROC	Receiver operating characteristic
